# Inflamm-Aging of Hematopoiesis, Hematopoietic Stem Cells, and the Bone Marrow Microenvironment

**DOI:** 10.3389/fimmu.2016.00502

**Published:** 2016-11-14

**Authors:** Larisa V. Kovtonyuk, Kristin Fritsch, Xiaomin Feng, Markus G. Manz, Hitoshi Takizawa

**Affiliations:** ^1^Division of Hematology, University Hospital Zurich, University of Zurich, Zurich, Switzerland; ^2^International Research Center for Medical Sciences, Kumamoto, Japan

**Keywords:** ageing, inflammation, hematopoietic stem cell, niche, cytokine, pathogen recognition receptor

## Abstract

All hematopoietic and immune cells are continuously generated by hematopoietic stem cells (HSCs) and hematopoietic progenitor cells (HPCs) through highly organized process of stepwise lineage commitment. In the steady state, HSCs are mostly quiescent, while HPCs are actively proliferating and contributing to daily hematopoiesis. In response to hematopoietic challenges, e.g., life-threatening blood loss, infection, and inflammation, HSCs can be activated to proliferate and engage in blood formation. The HSC activation induced by hematopoietic demand is mediated by direct or indirect sensing mechanisms involving pattern recognition receptors or cytokine/chemokine receptors. In contrast to the hematopoietic challenges with obvious clinical symptoms, how the aging process, which involves low-grade chronic inflammation, impacts hematopoiesis remains undefined. Herein, we summarize recent findings pertaining to functional alternations of hematopoiesis, HSCs, and the bone marrow (BM) microenvironment during the processes of aging and inflammation and highlight some common cellular and molecular changes during the processes that influence hematopoiesis and its cells of origin, HSCs and HPCs, as well as the BM microenvironment. We also discuss how age-dependent alterations of the immune system lead to subclinical inflammatory states and how inflammatory signaling might be involved in hematopoietic aging. Our aim is to present evidence supporting the concept of “Inflamm-Aging,” or inflammation-associated aging of hematopoiesis.

## Introduction

### Hematopoiesis

Hematopoiesis is an active, continuous process involving the production and consumption of mature blood cells that constitute the hemato-lymphoid system. It has been estimated that in a healthy individual (70 kg), approximately 5 × 10^11^ mature blood cells are produced daily throughout that individual’s lifetime (1). All blood cells arise from a small population of hematopoietic stem cells (HSCs) in the bone marrow (BM) that have two unique properties: self-renewing capacity, the ability to generate themselves, and multi-lineage differentiation capacity, the ability to produce all blood cell types, including red blood cells, platelets, myeloid lineage cells, such as monocytes and granulocytes, as well as lymphoid lineage cells, such as natural killer (NK) cells, B cells, and T cells. Since, in the steady state, most adult HSCs are in the G0 phase of cell cycle, i.e., they are quiescent and are estimated to turnover slowly on a monthly time scale (2–5), daily hematopoietic production is mainly sustained by highly proliferative downstream hematopoietic progenitor cells (HPCs) (6–8). Cellular behavior of HSCs, i.e., self-renewal, differentiation, and apoptosis, is tightly controlled by both cell-intrinsic factors, e.g., transcriptional regulatory networks and cellular metabolism, and cell-extrinsic factors, e.g., cytokines, chemokines, growth factors, metabolites, and exogenous pathogen-derived molecules (9–11). When mature hematopoietic cells are consumed and need to be replenished in response to hematopoietic challenges, HSCs can translate locally produced and/or systematically migrating external signals into hematopoiesis by increasing their own proliferation and differentiation (12, 13).

### Hematopoietic Stem Cell Heterogeneity

**Figure 1:**
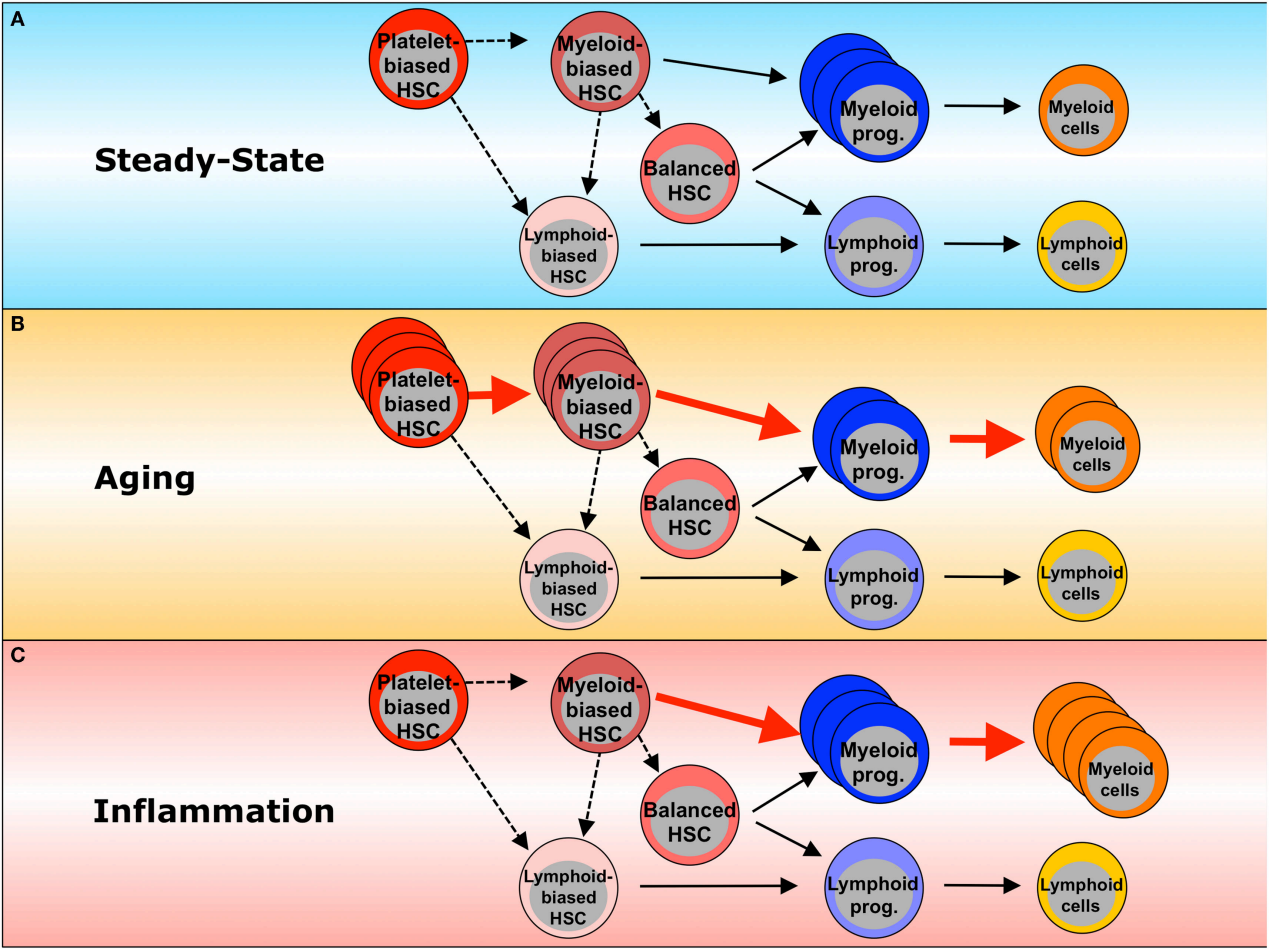
**Inflammation- and aging-associated changes in hematopoiesis**. **(A)** In steady state, platelet-biased HSCs are at the top of the hematopoietic hierarchy and are able to generate myeloid-biased and lymphoid-biased HSCs. In turn, myeloid-biased HSCs can generate both balanced- and lymphoid-biased HSCs, whereas lymphoid-biased HSCs do not generate their myeloid-biased counterparts. Platelet-biased HSCs have the potential to repopulate platelet populations faster than other HSC subsets. Myeloid-biased HSCs preferentially give rise to myeloid lineage cells through myeloid committed progenitors. Balanced HSCs make equal contributions to both myeloid and lymphoid lineages. Lymphoid-biased HSCs predominantly generate lymphoid over myeloid lineage cells through lymphoid-committed progenitors. Dashed lines represent the potential of one HSC subset to generate another HSC subset. Solid lines represent differentiation potential. **(B)** Inflammation enhances myeloid lineage production, including myeloid progenitors and mature myeloid cells, leading to myeloid bias in hematopoiesis. **(C)** During the processes of aging, myeloid-biased HSCs increase and produce more myeloid than lymphoid cells. Red arrows indicate the dominant differentiation pathway. Dashed lines represent a potential pathway. Solid lines represent the differentiation potential shown previously. The thickness of the lines reflects the relative contributions to each lineage commitment.

Recent studies with single-cell transplantation and lineage tracing have revealed cellular heterogeneity within a functionally defined HSC population and have identified HSC subtypes with distinct lineage differentiation potentials (Figure 1A) (14–18). Myeloid-biased HSCs give rise to more myeloid lineage than lymphoid lineage cells, while lymphoid-biased HSCs favor lymphopoiesis over myelopoiesis, and balanced HSCs produce myeloid and lymphoid lineage cells equally (14, 16). It has also been indicated that there is a certain functional hierarchy within these lineage-biased HSCs (16, 19). Myeloid-biased HSCs defined as CD150^high^CD34^−^ LKS have greater self-renewal potential than lymphoid-biased or balanced HSCs (CD150^low/negative^CD34^−^ LKS) and are able to replenish all types of HSC populations, indicating that myeloid-biased HSCs are higher in the HSC hierarchy than other HSCs. In addition, it was recently found that a subset of HSCs expressing von Willebrand factor efficiently gives rise to platelets and erythroid lineage cells earlier than other lineages, and this subset was therefore named platelet-biased HSCs. As the platelet-biased HSCs can generate myeloid-biased HSCs, they are suggested to be positioned at the apex of the hematopoietic hierarchy (17, 20).

Cellular heterogeneity and lineage priming have been observed at the level of not only HSCs but also HPCs (6, 21–23). A subset of multipotent progenitors (MPPs), MPP2 (LKS Flt3^−^CD150^+^CD48^+^), preferentially gives rise to platelets, erythrocytes, and granulocyte-monocyte progenitors (GMPs), while MPP3 (LKS Flt3^−^CD150^−^CD48^+^) is primed to GMP, and to a lesser extent, the erythroid lineage with no potential for platelet generation. MPP4 (LKS Flt3^+^CD150^−^CD48^+^) is biased toward lymphoid lineage output. These MPP subsets are independent of one another and, as each is produced directly by HSCs, they lack the capacity to give rise to one another. However, it remains to be determined whether HSC subsets with distinct lineage outputs have any clonal relationship with MPP subsets.

## Hematopoietic Aging

### Hematopoietic Changes during Aging

**Figure 2:**
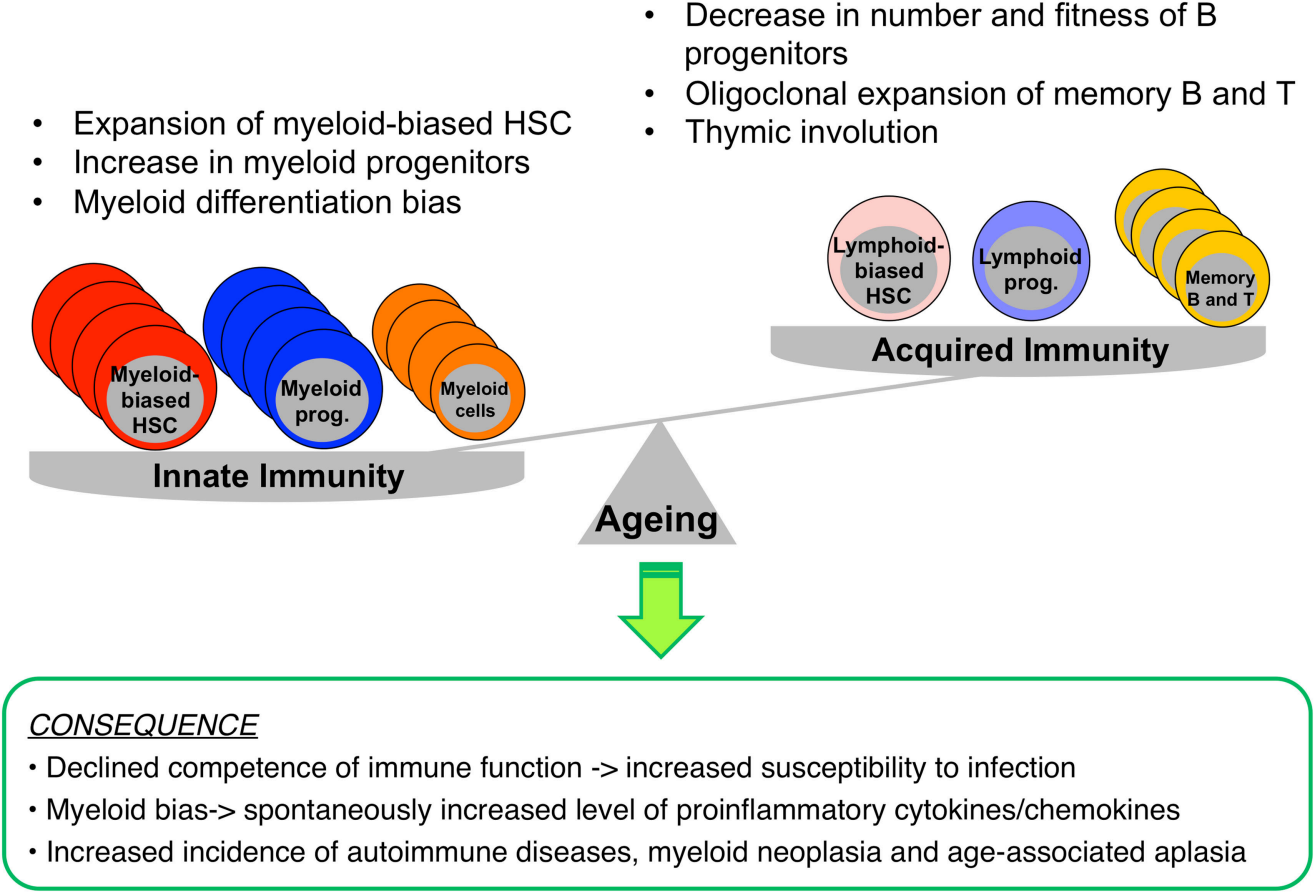
**Impacts of aging on immunity and hematopoiesis**. During aging, myelopoiesis results in the domination of hematopoiesis over lymphopoiesis due to increased numbers of myeloid-biased HSCs, myeloid progenitors, and myeloid cells, while the pool consisting of B and T cells shrinks. These hematopoietic changes result in increased dependence of the immune system on innate rather than acquired immunity, with an enhanced basal level of inflammation, increasing the risk of myeloid neoplasia or spontaneous anemia.

Aging of the hematopoietic system is represented by functional declines in both the adaptive and the innate immune system, an immunosenescence that leads to high susceptibility to infections, low efficacy of vaccinations, and increased vulnerability to the development of autoimmunity and hematologic malignancies (24, 25). As shown in Figure 2, (a) B cell production decreases significantly with advancing age, i.e., the naïve B cell pool diminishes, while the memory B cell pool expands. Diversity of the B cell repertoire also decreases in association with lowered antibody affinity and impaired class switching. B cells are prone to produce auto-antibodies increasing the incidence of spontaneous autoimmunity (26, 27); (b) *de novo* T cell production also declines with aging partially due to thymic involution. CD8^+^ T cells undergo oligoclonal expansion and their repertoire is skewed toward previously encountered antigens, as niches for naïve T cells in peripheral lymphoid tissues become occupied by terminally differentiated cells (28); (c) NK cells show diminished cytotoxicity and cytokine secretion; (d) although myeloid cells increase in number, their functionality is decreased, e.g., neutrophils migrate less in response to stimuli, and macrophages have reduced phagocytic activity and decreased oxidative burst (29–31); and (e) erythropoiesis also declines in elderly people causing frequent anemia (32), while the thrombocytic lineage has not, to date, been reported to be significantly affected by aging.

### HSC Functional Alteration during Aging

Since multiple blood lineages change during the aging process, it is possible that hematopoietic aging is in part due to functional changes in early hematopoietic compartments that repopulate the affected lineages, including HSCs. Single-cell and limiting dilution transplantations have demonstrated the self-renewal capacity of HSCs to apparently be reduced on a per-cell basis during aging, as the frequency of phenotypically defined HSCs does not correlate with that of functionally defined HSCs in aged BM (19, 33–38). It was also shown that phenotypic HSCs (LKS CD34^−^Flt3^−^) upregulate CD150 expression (19, 37, 39), resulting in expansion of the myeloid-biased HSC population and the domination of this fraction over the entire aged HSC pool (39), with advancing age (Figure 1B). Consistent with the phenotypic characterization, hematopoietic repopulation after transplantation is biased toward myeloid cell production, and this change in differentiation potential persists over the course of serial transplantations, indicative of aging-associated cell-autonomous alterations in HSCs. Based on these observations, two possible theories for age-associated myeloid bias can be proposed: (a) clonal evolution within the aged HSC population, in which lymphoid-biased HSC clones turn into myeloid-biased or platelet-biased HSC clones *via* cell-intrinsic changes (40); (b) clonal composition shift, in which subsets of myeloid-biased or platelet-biased HSC clones dominate the entire HSC pool *via* clonal expansion and/or selection (16, 19, 34, 39, 41–43). Aging-associated myeloid lineage skewing may also involve disturbance in the composition of committed progenitors: aged mice show a decreased frequency of common lymphoid progenitors, while frequencies of GMPs are increased (37). These findings are accompanied by decreased B cell lymphopoiesis and diminished fitness of lymphoid progenitors, coinciding with altered receptor-associated kinase signaling (44). Moreover, the recent identification of myeloid-restricted progenitors with long-term repopulating capacity/self-renewal has raised new questions regarding the definition of HSCs (18, 45). Therefore, which level of the hematopoietic hierarchy is affected by aging remains uncertain.

The BM homing efficiency of aged HSCs is significantly reduced when transplanted intravenously into irradiated recipients (14), although similar mobilizing efficacies are observed in aged and young HSCs released into the circulation in response to granulocyte colony-stimulating factor (G-CSF) treatment (46). Transcriptome profiling of aged versus young HSCs has provided molecular insights into potential mechanisms of HSC aging (33, 47): aged HSCs show dysregulation of intracellular homeostasis, e.g., upregulated stress responses, increased pro-inflammatory signaling, protein misfolding, downregulated DNA repair machinery, and aberrant chromatin modification (19, 33, 37). Further investigations have demonstrated that aged HSCs accumulate more DNA damage possibly due to higher levels of intracellular reactive oxygen species (ROS) and naturally produced genotoxic metabolites (48–50), but interestingly, these cells are still able to efficiently repair the damage upon cell cycle induction (51). Other studies have indicated that accumulation of proliferative stress in aged HSCs causes inefficient DNA replication and transcriptional repression (52). Aged HSCs also exhibit activation of the mammalian target of rapamycin (mTOR) (53), autophagy-dependent survival (54), dysregulated DNA methylation, specifically at the site of genes controlling myeloid and lymphoid balancing (51), impaired histone modification (55), and disturbed cell polarity (56).

These characteristics of HSC aging can, in part, be experimentally recapitulated by increasing the proliferative history of HSCs or stressing them with multiple injections of myeloablative chemotherapeutic regimens (57), or by conducting serial transplantations (“experimental aging”) (34, 35). As this indicates that proliferative history might be associated with the aging process, several groups have compared the cycling activity of young versus aged HSCs. The results are, however, controversial: some data indicate that aged HSCs have increased cycling activity (36), whereas others suggest no difference in cell cycle status (33, 38), or more quiescent HSCs in aged as compared to young BM (4, 53). These seemingly discrepant results might be partially explained by differing immunophenotypic definitions of HSCs and/or different experimental approaches to measure cell cycle status [reviewed in Ref. (3)].

### Aging-Associated Changes in BM Niche

Hematopoietic stem cell homeostasis is preserved in the BM microenvironment, the so-called HSC niche that supplies these cells with pivotal factors for their own maintenance (3, 58). Recent research on the BM niche has revealed a perivascular HSC niche comprised mesenchymal stromal cells (MSCs) and endothelial cells (ECs) as major cellular components, reflecting hierarchic HSC function and the effects exerted by aging (58, 59). MSCs are characterized by plastic adherence, high growth potential, and mesenchymal immunophenotypes, as well as differentiation into mesenchymal lineages, such as osteocytes, adipocytes, chondrocytes, fibroblasts, and epithelial cells (60, 61). Aged MSCs exhibit reduced clonogenic and proliferative capacity, and differentiation potentials are skewed toward adipogenesis at the expense of osteogenesis (62–64). These cells also show enlargement, telomere shortening, or p53/p21-mediated DNA damage accumulation, impaired DNA methylation or histone acetylation, and increased levels of ROS and nitric oxide (NO) (65–69). Although age-dependent mechanisms underlying adipogenesis-favoring MSC differentiation are not as yet fully understood, possible molecular changes have been reported, including activations of peroxisome proliferator-activated receptor gamma 2 and CCAAT/enhancer binding protein (70, 71). Adipogenesis enhancement in aged BM (72) might be linked to dysregulation of insulin growth factor signaling (27), changes in extracellular matrix composition, and decreased bone formation (73, 74). Since adipocytes are shown to negatively regulate HSC function and B-lymphopoiesis (75, 76), adipogenesis enhanced in aged BM might promote myelopoiesis over lymphopoiesis as well as impair HSC function. In fact, young HSCs in the aged environment reportedly tend to produce slightly more myeloid cells than in a young environment (37, 77).

Endothelial cells are another niche cell component that secrete HSC maintenance and retention factors, such as stem cell factor and CXC motif ligand (CXCL) 12 (58, 78). Aging involves decreases in CD31^hi^Emcn^hi^ EC-associated osteoprogenitors (79), fewer PDGFRβ^+^/NG2^+^ perivascular cells, arterioles, and ECs, thereby resulting in reduced stem cell factor production (59). Activation of endothelial Notch signaling can reverse these age-dependent vascular niche alterations, without affecting aged HSC function. Additionally, vascular endothelial function declines with aging, due to reduced NO which in turn induces vasodilation, elevated oxidative stress causing genomic instability, and increased ROS levels associated with impaired proangiogenic functions of EC (80). As it has been suggested that NO production regulates CXCL12-mediated HSC mobilization (81), aging-related reductions of EC-derived NO and the enhancement of angiogenic function in the BM niche might be involved in aberrant HSC maintenance and/or retention in aged BM.

### Hematopoietic Aging in Humans

While most of the data on aging of the hematopoietic system was obtained employing a mouse system, a few pioneering studies have indicated similar tendencies in the human hematopoietic system. HSCs containing fractions such as Lin^−^CD34^+^CD38^−^ (29), Lin^−^CD34^+^CD38^−^CD90^+^CD45RA^−^ (82), or Lin^−^CD34^+^CD10^−^CD123^−^CD45RA^−^CD90^+^ (83) increase with age. While GMPs appear to be retained at the same frequency (29, 82, 83), early B cell progenitors and CLPs decrease with advancing age (82, 83). Yet, the functionality and differentiation bias of HSCs remain unclear: one study, using xenograft mouse models, indicated no change in NSG-repopulating cell frequency and decreased myeloid lineage repopulation of aged HSCs (29), while another (82) showed a two-fold decreased engraftment with significant myeloid lineage dominance. Further molecular analyses indicated upregulations of myeloid and megakaryocyte-associated genes and downregulations of lymphoid differentiation genes (82, 83). These findings indicate major aging-associated changes in hematopoiesis to be conserved among species.

## Inflammation in Hematopoiesis

### Hematopoietic Responses to Inflammation

Inflammation is defined as a protective immune response, underlain by a variety of pathophysiological processes that are in part caused by infection and tissue injury/damage (84). There are many different types of endogenous and exogenous factors that can potentially induce local or systemic inflammation: mechanical stimuli (e.g., tissue damage, foreign objects), thermal stimuli (e.g., heat, cold), radiation (e.g., ultraviolet, ionizing, chemotherapeutic), chemical irritants (e.g., acids, alkalis, toxins), physical or psychological stress, autoimmunity (e.g., allergens, autoantigens), and pathogens (e.g., bacteria, viruses, fungi, protozoal parasites).

Inflammatory responses involving the hemato-immune system are exemplified by infection. The first line of defense against infection is often initiated by innate immunity: bacterial, viral, or fungal pathogens that break through the epithelial barrier will be recognized by pattern recognition receptors (PRRs) expressed on hematopoietic and non-hematopoietic cells, such as dendritic cells (DCs), macrophages, monocytes, and ECs (85). Upon ligation of PRR by pathogen-derived molecules, the innate immune cells secrete an array of inflammatory cytokines and chemokines, e.g., interleukin (IL)-1β, IL-6, CXCL-8, IL-12, and tumor necrosis factor (TNF)-α, as well as chemical mediators, e.g., leukotriene B_4_, prostaglandin E_2_ (PGE2), and histamine [reviewed in Ref. (86, 87)]. These biologically active factors attract immune effector cells from the circulation to the site of infection, and simultaneously increase vascular permeability to allow more immune effector cells to infiltrate and differentiate in the inflamed tissues. This process causes heat (calor), pain (dolor), redness (rubor), and swelling, which are referred to as hallmarks of inflammation (87).

Toll-like receptors (TLRs) belong to the PRR family and recognize microbial products derived from exogenous pathogen molecules, such as lipopolysaccharide (LPS) for TLR-4, lipopeptides for TLR-1, -2, and -6, bacterial and viral RNA and DNA for TLR-3, -7, -8, and -9, as well as possibly endogenous host molecules that are misfolded or modified, including heat shock proteins and fibronectin. Binding of the respective ligands to TLRs leads to cell proliferation, differentiation, and migration (88). For example, activation of TLR signaling on DCs is a key step necessary for their full maturation into antigen-presenting cells. When TLRs on DCs are ligated by the pathogen molecules encountered, DCs enhance the subcellular machineries that process and present pathogen-derived antigens on their cell surfaces, as well as triggering the production of pro-inflammatory cytokines/chemokines, e.g., macrophage inflammatory protein (MIP)-1α, MIP-1β, and the regulated on activation, normal T cell expressed and secreted (RANTES) chemokine. They also up/downregulate chemokine receptors, e.g., CCR1, CCR5, and CCR7, thereby directing their migration to secondary lymphoid organs, such as lymph nodes or the spleen, where they initiate a second-line defensive response by activating adaptive immunity [reviewed in Ref. (89)]. Naïve T cells are activated to proliferate and differentiate into functionally mature effector cells upon activation of two distinct signals through antigen-presenting cells expressing the major histocompatibility complex and co-stimulatory molecules. These effector T cells in turn migrate to the site of infection, and either activate other immune effector cells (macrophages, NK cells, neutrophils, eosinophils, mast cells, basophils) through cytokine secretion or kill the infected cells through release of cytolytic molecules. They also produce IL-4, IL-5, interferon (IFN)-γ, and tumor growth factor (TGF)-β to activate monoclonal B cells that recognize the specific antigen. This activation process facilitates clonal expansion and differentiation into plasma and memory B cells that secrete high-affinity antibodies, and therefore enhances subsequent humoral immune responses, e.g., neutralization, phagocytosis, and opsonization.

### HSC Response to Infection

**Figure 3:**
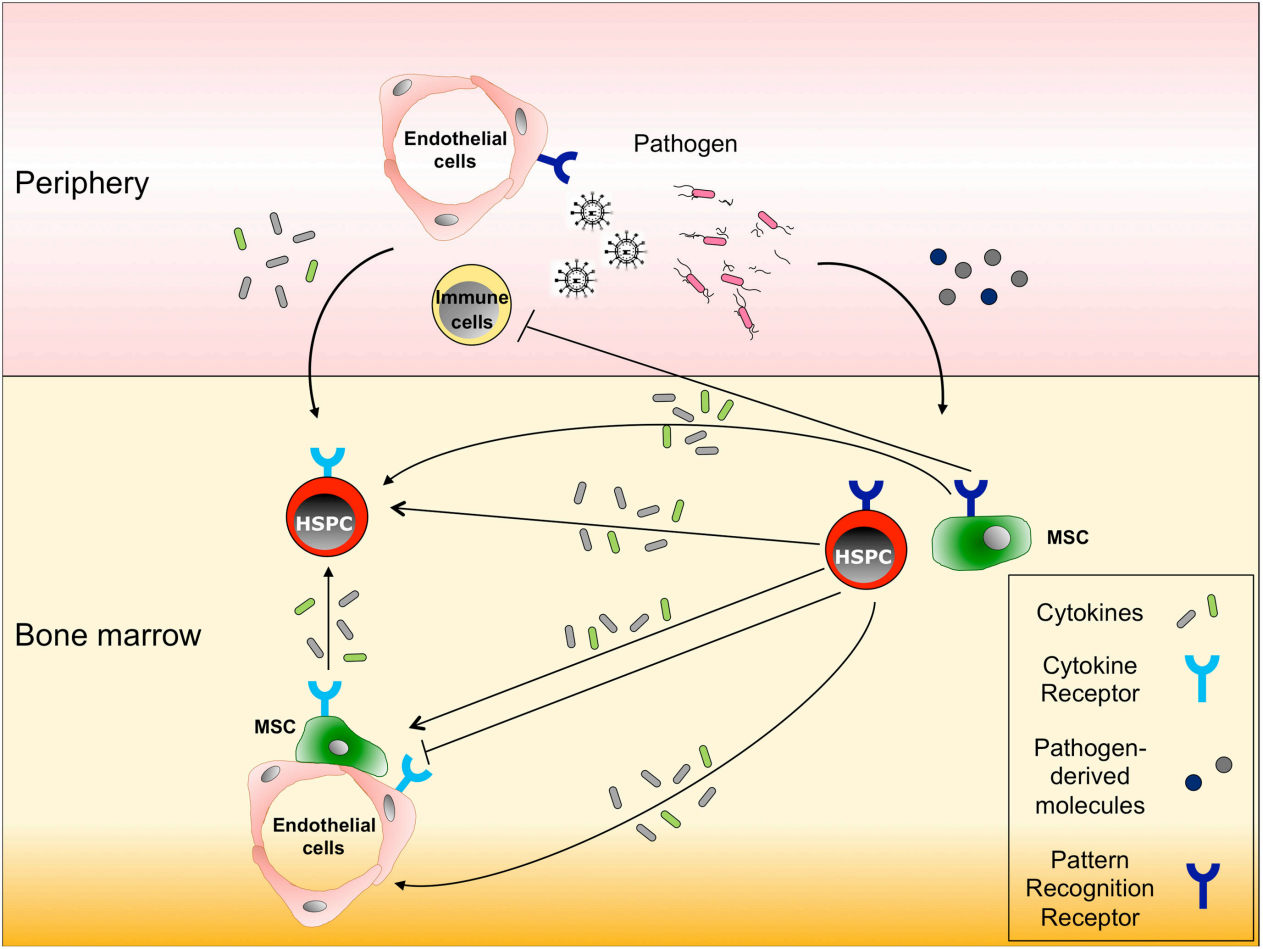
**Responses of hematopoietic stem and progenitor cells (HSPCs), and non-hematopoietic bone marrow cells to infection**. HSPCs and non-hematopoietic cells in bone marrow (BM), such as mesenchymal stromal cells (MSCs) and endothelial cells, express both cytokine and pattern recognition receptors (PRRs) on their surfaces. In response to infections in peripheral tissues, (a) immune cells or endothelial cells secrete pro-inflammatory cytokines that migrate to the BM and stimulate the respective receptors expressed on HSPCs, thereby inducing their proliferation, migration and/or differentiation; (b) alternatively, migrating cytokines also can act on MSCs or endothelial cells to enhance their pro-inflammatory cytokine production; and (c) some pathogen-derived molecules reach the BM and activate HSPCs directly through PRRs or indirectly through pro-inflammatory cytokines produced by PRR expressing MSCs or HSPCs.

Since immune effector cells involved in both innate and adaptive immunity are short-lived except for the memory B and T cells maintained for life, they need to be replenished by the upstream HSCs and progenitor cells (HSPCs) in BM when consumed during inflammation (12). Given that HSCs tend to remain in a largely quiescent state, as mentioned above (5), understanding how these quiescent HSCs respond to inflammation and are activated to maintain hematopoietic homeostasis is becoming a major research focus. Recent findings have indicated that not only mature immune cells but also HSPCs are capable of responding to infection by directly sensing pathogen-associated molecule patterns through their respective PRR (4, 13, 90–92) (Figure 3). The activation of PRR in HSPCs leads to enhanced proliferation, increased mobilization from the BM, reduced self-renewal, and myelopoiesis-favoring differentiation (Figure 1C). Since HSPCs also express a broad spectrum of inflammatory cytokine/chemokine receptors (12, 93), they can detect milieu broad range of pro-inflammatory signals *via* their respective receptors, released systemically or locally by activated immune cells in response to infectious challenges, e.g., IFN-α/γ (94–98) (Figure 3). These two pathways are not mutually exclusive: G-CSF stimulation impairs HSC repopulating potential through upregulation of TLR-2 and -4 expressions and activation of the subsequent signaling in HSCs (99). When bound to TLR-2 and -4, HSPCs have been shown to have the capacity to secrete pro-inflammatory cytokines, e.g., IL-6, TGF-β, TNF-α, and granulocyte-macrophage colony-stimulating factor (GM-CSF), all of which activate their respective receptor signaling mechanisms and promote myelopoiesis in a paracrine or an autocrine fashion (13). The HSPC responses triggered by TLR activation appear to integrate the infectious signals into the process of hematopoiesis by recruiting themselves to the inflamed tissues, directing their differentiation, and reflecting the high demand toward myelopoiesis. A recent study identified stem cell-like megakaryocyte-committed progenitors as a novel inflammation responsive cell population remaining dormant and thereby show little contribution to megakaryopoiesis in the steady state, but in response to acute inflammation, these cells become metabolically active and rapidly produce platelets to replace those lost during inflammatory processes (45).

### BM Niche Response to Infection

MSCs can influence both innate and acquired immunity through cell–cell contact or secretion of soluble factors, e.g., TNF, IL-10, IL-6, and PGE2 (100), which inhibit T cell function, DC maturation, and the activation and proliferation of both B and NK cells. MSCs have also been shown to express several TLRs, the activation of which controls inflammatory cytokine production critical for their immunosuppressive function (101, 102). TLR activation in MSCs reportedly not only influences their functions, e.g., differentiation, proliferation, migration, immunomodulation, and bone regeneration (103–105), but also regulates HSPC proliferation and differentiation toward myeloid development, as well as monocyte egress (106, 107) (Figure 3). Moreover, it has been shown that pro-inflammatory cytokines control the productions of other cytokines/chemokines by MSCs. IFN-γ alone or in combination with TNF or IL-1 induces the production of nitric oxide synthase or PGE2 in MSCs, thereby inhibiting T or NK cell activation, respectively (108). The secretion of IFN-γ by cytotoxic CD8^+^ T cells also leads indirectly to an activation of HPCs by promoting the IL-6 production in MSCs (109). During early hematopoietic regulation, G-CSF suppresses the production of CXCL12 from BM MSCs and mobilizes HSC into circulating blood (110).

ECs also express multiple PRRs, and when activated, regulate various immune responses acting against infection [reviewed in Ref. (111)] (Figure 3). In response to TLR4 activation, ECs produce G-CSF that contributes to rapid neutrophil production in the BM (112, 113), and at the same time, induces neutrophil recruitment to infectious sites (114, 115). Pro-inflammatory cytokine signaling influences EC function through nuclear factor κB (NFκB) activity. It has also been shown that stimulation of ECs in the BM with TNF-α and LPS expands the HPC population through modulation of Notch signals (66). When stimulated with IL-1β and TNF-α, ECs in the BM are induced to produce GM-CSF, which leads to the recruitment of neutrophils and expansion of HPCs in the BM (116, 117). Interestingly, it was also shown that through MSC-EC interaction, MSCs upregulate IL-6 production modulating the responses of ECs to inflammatory cytokines (118). The results obtained in these studies suggest that ECs in the BM play important roles in hematopoietic regulation during inflammation.

Taken together, these findings highlight previously unappreciated HSPC responses to infection *via* TLR-mediated direct or cytokine-mediated indirect sensing mechanisms. Inflammatory responses play beneficial roles in the activation and replenishment of hemato-immune system components and thereby contribute to controlling infection. However, if the inflammatory response is not discontinued in a timely manner, instead being unnecessarily sustained even after infection has resolved, it might ultimately have detrimental effects, e.g., tissue damage, chronic diseases, and even cancer. In fact, uncontrolled persistent inflammatory signaling is known to promote the development of chronic diseases, e.g., rheumatoid arthritis, inflammatory bowel disease, asthma, and aplasia. Recent studies have also demonstrated that sustained IFN-α/γ activation during chronic infection impairs HSC function and can ultimately lead to BM failure (94, 119). Chronic TNF-α signaling is associated with myelodysplastic syndrome and BM failure (120). Therefore, inflammatory responses must stop when no longer needed. The appropriate timing of this cessation is critical.

### Inflammation of the Hematopoietic System in Humans

Not only murine but also human HSCs have been shown to express TLRs (121, 122). The stimulation of agonists for TLR2, TLR7, and TLR8 *in vitro* induces cytokine production, e.g., IL-1b, IL-6, IL-8, TNF-α, and GM-CSF, as well as cell differentiation of the myeloid lineage (122, 123). *In vitro* TLR9 binding by CpG DNA reportedly upregulates IL-8 expression *via* activation of ERK1/2 and p38, both mitogen-activated protein kinases, but not of NFκB (121). Nevertheless, in contrast to murine HSCs, quiescent human HSCs appear to be fully resistant to infection with both intracellular bacteria, such as *Listeria monocytogenes* and *Salmonella enterica*, and extracellular bacteria, including *Yersinia enterocolitica* (124). In contrast, when human CD34^+^ HSPCs were cultured with *Escherichia coli*, they showed upregulated production of pro-inflammatory cytokines such as IL-1, IL-6, IL-8, and TNF through NFκB activation, as do human CD34^+^-derived granulocyte–macrophage lineage cells (125). Regarding the HSPC response to inflammatory cytokines, the exposure of human CD34^+^ HSPCs to IFN-γ produces drastic transcriptional changes in genes involved in pro-apoptotic processes, immune responses, and myeloid proliferation that results in an increased number of viable cells (126, 127). While some transcriptional changes are specific to HSPCs, others, e.g., cell growth and signal transduction, generally occur in stromal cells incubated with IFN-γ (127). In contrast, *in vitro* stimulation with IFN-γ and TNF severely compromised the ability of HSPCs to undergo multi-lineage reconstitution in xenografted mice (128, 129). Future studies are required to unravel the details of the different HSPC responses to inflammatory cues and the underlying molecular mechanisms.

## Aging-Associated Inflammation: *“Inflamm-Aging”*

There are similarities between hematopoietic alterations during inflammation and those that occur with aging. In response to aging and bacterial infection, myelopoiesis becomes dominant over lymphopoiesis in relation to immunosenescence (36, 38, 130, 131). Most notably, B-lymphopoiesis is impaired due to a decreased level of E47, a transcription factor essential for B cell development, in aged (132) and LPS-treated mice (130). The aging-associated myeloid dominance and/or adipogenesis in BM might be triggered by increased basal levels of pro-inflammatory cytokines even in the absence of infection. Indeed, levels of circulating pro-inflammatory cytokines, such as IL-6, TNF-α, IL-1Rα, and C-reactive protein, are reportedly upregulated in healthy elderly populations (25, 133–138). These observations allow us to hypothesize that “Inflamm-Aging” represents a subclinical grade of chronic inflammation possibly contributing to the initiation and/or acceleration of hematopoietic aging.

Aging-associated HSC alterations, including reduced self-renewal and myelopoiesis-favored differentiation, are very similar to the functional changes occurring in HSCs exposed to chronic inflammatory stimuli: the HSC pool is shifted to an increased proportion of CD150^high^ HSCs that predominantly produce myeloid lineage cells, as mentioned earlier (19, 39). This was also the case when mice were given a daily low dose of LPS for 1 month, suggesting exogenous stimulation to be involved in HSC aging (130). Recent studies have indicated that accumulation of myeloid-skewed HSCs with aging is mediated by activated signaling of TLR4 (130), P-selectin (33), NFκB (33), the RANTES-mTOR pathway (53, 77), and TGF-β (19). Since numerous pro-inflammatory cytokines are known to be produced by myeloid cell lineages or adipocytes, there might be a positive feedback mechanism by which, when aging processes begin, HSCs with myeloid-skewed differentiation gradually accumulate in BM *via* proliferative signals. As a consequence, these cells give rise to more myeloid cells that are prone to spontaneously produce pro-inflammatory cytokines, thereby further advancing myeloid dominance (Figure 2). In parallel, adipogenic MSC differentiation during aging also contributes to the promotion of inflammatory cytokine production and enhanced myelopoiesis. Moreover, inflammatory conditions foster the production and release of ROS in hematopoietic cells, a genotoxic reagent known to damage DNA, and might cause genetic ablations in adjacent cells, such as MSCs and ECs in BM (48). Intracellular ROS production has indeed been shown to be induced and to cause DNA damage accumulation in HSCs during viral infections and aging (49, 119), although another study detected no DNA damage in aged HSCs (52).

## Discussion

**Figure 4:**
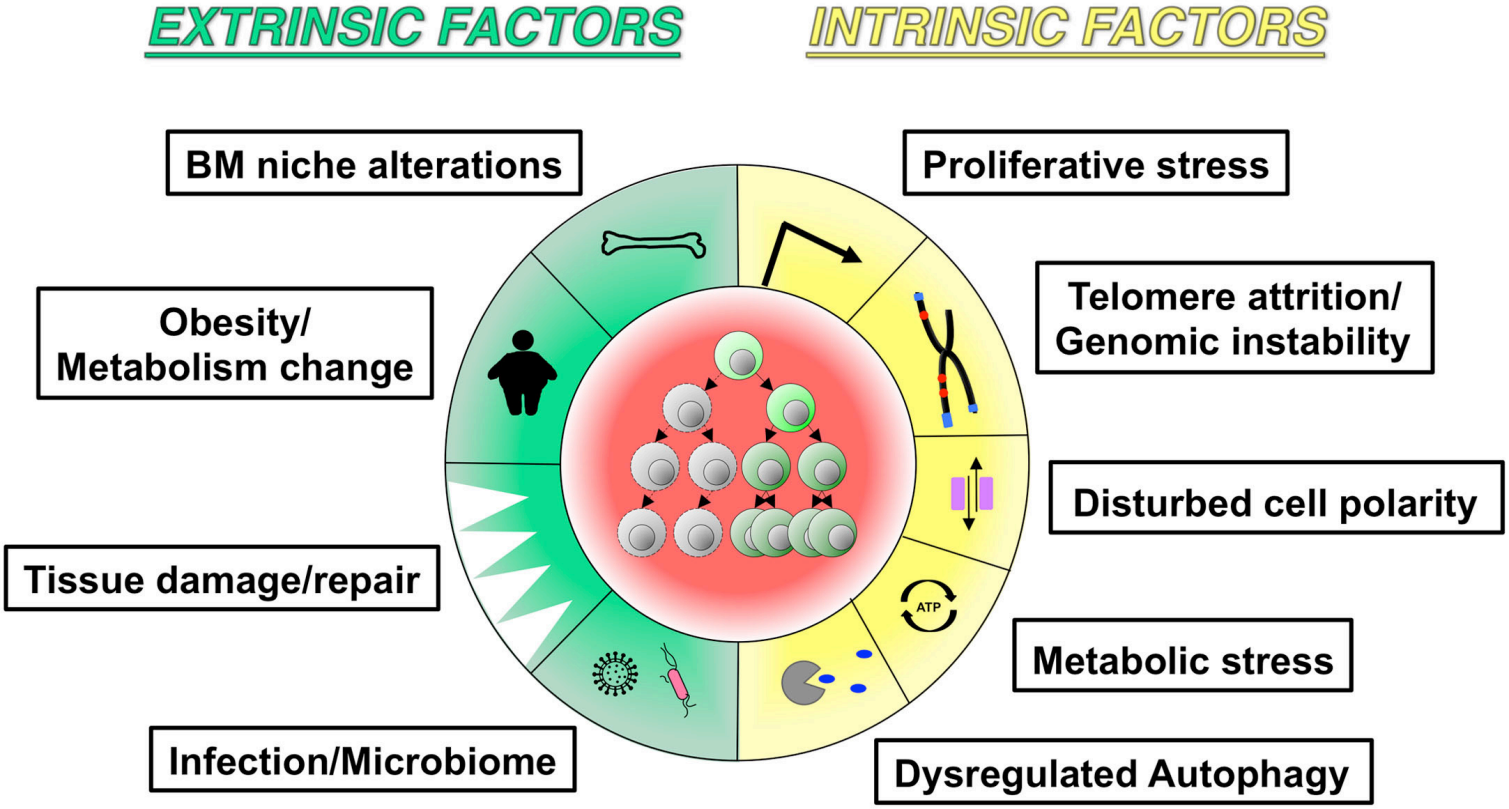
**Potential hallmarks of inflamm-aging**. The schematic figure highlights similarities between aging and inflammation-associated changes in hematopoiesis and hematopoietic stem cells: (a) extrinsic factors include bone marrow niche alterations, metabolic changes, tissue damage/repair, and infection/microbiome; (b) intrinsic factors include proliferative stress, telomere attrition/genomic instability, dysregulated autophagy, metabolic stress, and dysregulated autophagy.

Since numerous inflammatory factors are increased in aged hematopoietic tissues, and inflammation- and aging-associated hematopoietic changes share common cellular and molecular alterations, it is reasonable to speculate that low-grade inflammation might be involved in hematopoietic aging with reduced fitness of both adaptive and innate immune cells. Given that some hematopoietic phenotypes during inflammation and aging arise from functional alterations in HSPCs, as discussed above (19, 34, 39), it would be worthwhile to elucidate the underlying common mechanisms. Future research could yield meaningful insights into cell-intrinsic changes in HSPC quantity and quality, e.g., how aspects of HSPC population dynamics such as functional heterogeneity and population size change, whether all subsets of HSCs with a distinct lineage output respond equally to inflammatory stimuli or only the minor fraction is responsive, how the self-renewal and differentiation capacities of HSC are altered on a per-cell basis, and molecular changes in cellular signaling, such as alterations in cellular metabolism, transcriptional networks, epigenetic modifications, and genomic instability (Figure 4). It is also essential to understand to what extent inflamm-aging-associated cell-extrinsic factors influence HSPC biology, including signals derived from the BM niche, tissue damage/repair, infection, obesity, or the microbiome. In addition, the fundamental task that remains is identification of the factor(s) initially triggering the process of hematopoietic inflamm-aging. Inflammation- or aging-related external stimuli appear to force quiescent HSCs to proliferate and impair their self-renewal and differentiation capacities, as suggested by evidence that HSC cycling in response to chemotherapy administration or hematopoietic stress accelerates the manifestation of aging phenotypes (119). These data suggest that the central features of HSCs aging might be attributable to accumulation of a proliferative history that is closely associated with perturbed self-renewal and differentiation.

The incidence of myeloid leukemia increases with aging (139). It was recently demonstrated that age-related clonal hematopoiesis with somatic mutations is a risk factor for hematopoietic malignancies (140–144). Inflammation has long been regarded as a driving force for cancer development in many tissues (145). Indeed, high resolutional sequencing of human hematologic malignancies have revealed somatic mutations in different inflammatory signaling genes, such as NFκB, myeloid differentiation primary response gene 88, TLR4, and TNF receptor-associated factors, all of which result in hyperproliferation or prolonged survival of tumor cells (146). These findings imply a critical role of inflammatory signaling as a driving force for leukemogenesis. It would be interesting to investigate how chronically sustained inflammatory stimuli are involved in the acquisition of genetic ablations in pre-leukemic clones and the roles of clonal evolution in the development of hematologic malignancies. It may also merit mention that no mutations in inflammatory signaling cascades have been found in myeloid malignancies, raising the possibility of a protective mechanism functioning to prevent malignant myeloid transformation of HSCs.

Inflammation and aging have thus far been seen as two independent pathophysiological processes. However, a growing body of evidence has highlighted biological changes in hematopoiesis and HSCs that are common to both inflammation and aging. Thus, it is likely that sustained inflammatory stimuli contribute to hematopoietic aging and possibly leukemogenesis, supporting the inflamm-aging concept. Since inflammation and aging might both be involved in increased risk for leukemogenesis, eliminating unwanted inflamm-aging factors is a potential approach to preserving both HSC and immune functions, and thereby preventing a functional decline in hematopoiesis and the emergence of malignant clones. Future investigation is required to better characterize hematopoietic inflamm-aging processes at the tissue, cellular, and molecular levels.

## Author Contributions

LVK, KF, and XF wrote the manuscript and prepared the figures. MGM and HT wrote and critically revised the manuscript. All the authors approved the final version of this manuscript for submission.

## Conflict of Interest Statement

The authors declare that the research was conducted in the absence of any commercial or financial relationships that could be construed as a potential conflict of interest. The reviewer DL and handling editor declared their shared affiliation, and the handling editor states that the process nevertheless met the standards of a fair and objective review.
